# Tirofiban‐Associated Profound Thrombocytopenia in a Patient With Glucose‐6‐Phosphate Dehydrogenase Deficiency Undergoing Primary Percutaneous Coronary Intervention: A Case Report

**DOI:** 10.1155/cric/3617791

**Published:** 2026-05-13

**Authors:** Chuanwei Zhao, Yansi Wang, Shizhen Gao, Wei Meng, Yane Yang, Wenjie Tan

**Affiliations:** ^1^ Department of Cardiology, The Second People′s Hospital of Baoshan City, Baoshan, Yunnan, China; ^2^ Baoshan Traditional Chinese Medicine Hospital, Baoshan, China

**Keywords:** G6PD deficiency, heparin-induced thrombocytopenia, primary PCI, thrombocytopenia, tirofiban

## Abstract

**Background:**

Profound thrombocytopenia after tirofiban exposure during primary percutaneous coronary intervention (PCI) is rare but clinically important. Causal attribution may be challenging when unfractionated heparin is coadministered, and the relevance of glucose‐6‐phosphate dehydrogenase (G6PD) deficiency remains uncertain.

**Case Presentation:**

A 34‐year‐old man with known G6PD deficiency presented with an inferior ST‐segment elevation myocardial infarction and underwent primary PCI. He received aspirin, ticagrelor, and unfractionated heparin. Coronary angiography showed multivessel coronary disease with complete occlusion of the mid‐to‐distal right coronary artery and a large thrombus burden. After aspiration thrombectomy and drug‐eluting stent implantation, intracoronary tirofiban (2500 *μ*g bolus), followed by intravenous tirofiban infusion (0.15 *μ*g/kg/min), was administered as bailout antithrombotic therapy. Within 6 h, the platelet count fell from 207 × 10^9^ to 1 × 10^9^/L. Peripheral blood smear showed no platelet clumping or abnormal cells. Sepsis and disseminated intravascular coagulation were not supported by laboratory findings. Heparin‐induced thrombocytopenia was considered highly unlikely because the 4Ts score was 1, reflecting the extremely early onset, absence of new thrombosis, and the presence of a more plausible alternative explanation. The clinical course was therefore most consistent with acute tirofiban‐associated profound thrombocytopenia.

**Interventions and Outcomes:**

Tirofiban and heparin were discontinued immediately, and intravenous immunoglobulin (20 g/day) was administered. The platelet count recovered to 25 × 10^9^/L at 48 h, 48 × 10^9^/L at 72 h, and 124 × 10^9^/L on Day 4. Clopidogrel was restarted at 48 h, followed by indobufen at 72 h. No major bleeding or thrombotic complications occurred.

**Conclusions:**

This case supports tirofiban‐associated acute profound thrombocytopenia as the most likely diagnosis and underscores the importance of prompt drug withdrawal, structured differential diagnosis against heparin‐induced thrombocytopenia, and individualized antithrombotic reinitiation. The contribution of G6PD deficiency remains hypothetical and requires further study.

## 1. Introduction

Primary PCI is the preferred reperfusion strategy for ST‐segment elevation myocardial infarction (STEMI) [1]. Periprocedural antithrombotic therapy usually includes dual antiplatelet therapy and anticoagulation, whereas intravenous glycoprotein IIb/IIIa inhibitors are now generally reserved for bailout situations such as large thrombus burden, no‐reflow, or other thrombotic complications rather than routine use [2]. Acute thrombocytopenia is an uncommon but potentially serious adverse effect of glycoprotein IIb/IIIa inhibitors, including tirofiban [3]. In contrast, heparin‐induced thrombocytopenia (HIT) is a distinct immune‐mediated complication with different timing, pathophysiology, and management implications.

Glucose‐6‐phosphate dehydrogenase (G6PD) deficiency is the most common inherited enzymatic disorder of red blood cells and is associated with impaired cellular protection against oxidative stress [4]. Although its relevance to tirofiban‐associated thrombocytopenia remains uncertain, tirofiban‐related thrombocytopenia is a recognized, rare but potentially serious adverse effect of glycoprotein IIb/IIIa inhibitors [3, 5]. In acute coronary syndrome, this comorbidity may complicate antithrombotic decision‐making because treatment requires balancing ischemic benefit against bleeding and other hematologic adverse events.

We report a case of profound thrombocytopenia occurring shortly after tirofiban exposure during primary PCI in a patient with known G6PD deficiency. The purpose of this report is not to claim a proven mechanism, but to highlight the differential diagnosis, especially the distinction from HIT, and to describe an individualized strategy for temporary antithrombotic reinitiation.

## 2. Case Presentation

A 34‐year‐old man with a body mass index of 30.7 kg/m^2^, a 5‐year history of hypertension, and childhood‐diagnosed G6PD deficiency presented on December 14, 2024, with sudden severe back pain, diaphoresis, nausea, and radiation of pain to the left arm and lower jaw. Electrocardiography at the referring hospital showed ST‐segment elevation of > 0.2 mV in Leads II, III, and aVF, consistent with acute inferior STEMI.

Before transfer, the patient received aspirin 300 mg, ticagrelor 180 mg, and intravenous unfractionated heparin 5000 U. After arrival at our center, an additional 2000 U of unfractionated heparin was administered during the procedure, for a total periprocedural dose of 7000 U.

Coronary angiography showed severe multivessel coronary artery disease, including severe stenosis of the left anterior descending and left circumflex arteries and complete occlusion of the mid‐to‐distal right coronary artery with a large angiographic thrombus burden. Formal TIMI thrombus grading was not prospectively recorded. The patient underwent aspiration thrombectomy followed by drug‐eluting stent implantation in the right coronary artery. Because of the marked thrombus burden during primary PCI and the need for rapid parenteral platelet inhibition as bailout therapy, tirofiban was selected as an intensified intraprocedural antithrombotic strategy despite the background of G6PD deficiency, after individualized assessment of the immediate ischemic and hematologic risks [2].

The baseline platelet count was 207 × 10^9^/L. Six hours after the procedure, the platelet count dropped abruptly to 1 × 10^9^/L, indicating profound thrombocytopenia. Repeat monitoring confirmed persistence of severe thrombocytopenia during the early postexposure period. Peripheral blood smear showed no platelet aggregation or abnormal cells, making pseudothrombocytopenia unlikely. Procalcitonin was < 0.05 ng/mL, fibrinogen was 4.2 g/L, and D‐dimer was 0.8 mg/L fibrinogen equivalent units, arguing against sepsis or disseminated intravascular coagulation as the primary cause.

HIT was considered but judged highly unlikely. The 4Ts score was 1 because thrombocytopenia occurred within hours of periprocedural heparin exposure, there was no new thrombosis, and a more plausible alternative explanation was present [6, 7]. This very early onset was inconsistent with typical immune‐mediated HIT in a patient without known recent heparin exposure [6, 7]. Anti‐PF4/heparin antibody testing was not performed because the pretest probability of HIT was extremely low, and further immunoassay testing was unlikely to change diagnostic classification or management [6, 7]. Accordingly, the overall clinical picture favored acute tirofiban‐associated profound thrombocytopenia rather than a heparin‐mediated process.

**Figure 1:**
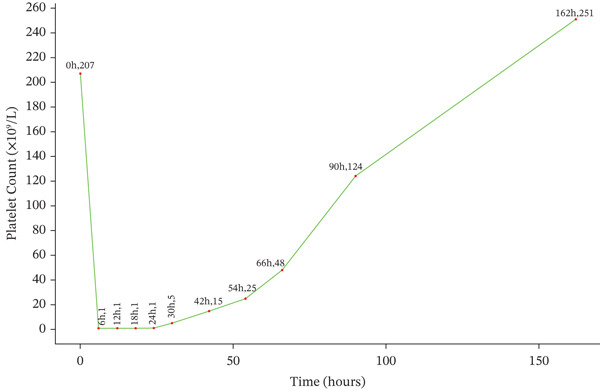
Temporal trend in platelet count after tirofiban exposure and treatment withdrawal.

All antithrombotic agents, including tirofiban and unfractionated heparin, were stopped immediately. Because of the profound platelet nadir and concern for bleeding risk after recent PCI, intravenous immunoglobulin (IVIG) (20 g/day) was administered. The platelet count rose to 25 × 10^9^/L at 48 h, and clopidogrel 75 mg/day was initiated. At 72 h, the platelet count increased further to 48 × 10^9^/L, and indobufen 100 mg twice daily was added as part of an individualized transitional regimen. By postoperative Day 4, the platelet count had recovered to 124 × 10^9^/L. No major bleeding or recurrent thrombotic events occurred during hospitalization. On postoperative Day 10, the patient was discharged with a stable platelet count of 348 × 10^9^/L, as shown in Figure 1.

## 3. Discussion

### 3.1. Differential Diagnosis of Profound Thrombocytopenia After Primary PCI

The principal diagnostic issue in this case was whether the profound thrombocytopenia was more likely related to tirofiban exposure or to concomitant heparin use. The overall clinical course favored acute tirofiban‐associated thrombocytopenia. Specifically, the platelet count fell profoundly within 6 h after tirofiban administration, a time course that has been repeatedly reported in acute tirofiban‐associated thrombocytopenia and in glycoprotein IIb/IIIa inhibitor‐related thrombocytopenia more broadly [3, 8]. By contrast, typical immune‐mediated HIT usually develops 5–10 days after heparin initiation unless there has been recent sensitizing heparin exposure [6, 7]. In addition, there was no new thrombosis or other clinical manifestation suggestive of HIT. The 4Ts score was 1, indicating a very low pretest probability, and the combination of extremely early onset, absence of thrombosis, and the presence of a more plausible alternative explanation argued strongly against a heparin‐mediated mechanism [6, 7].

In this context, anti‐PF4/heparin antibody testing would not be expected to materially change the diagnostic probability when the clinical pretest likelihood is extremely low [6, 7]. Pseudothrombocytopenia was excluded by peripheral blood smear, and sepsis, and disseminated intravascular coagulation were not supported by the available laboratory data. Taken together, these findings support acute tirofiban‐associated profound thrombocytopenia as the most plausible diagnosis in this patient.

From a clinical perspective, this case also underscores the importance of distinguishing acute glycoprotein IIb/IIIa inhibitor‐associated thrombocytopenia from HIT in patients undergoing PCI. Although both conditions may arise in the setting of combined antithrombotic exposure, they differ substantially in timing, presumed mechanism, and management implications [6–8]. Therefore, abrupt thrombocytopenia occurring within hours after tirofiban exposure should prompt early consideration of a glycoprotein IIb/IIIa inhibitor‐related reaction, whereas HIT should be assessed in the context of structured clinical probability rather than inferred solely from concurrent heparin use.

### 3.2. Potential Role of G6PD Deficiency in This Case

The clinical presentation in this patient is most consistent with acute tirofiban‐associated thrombocytopenia. However, the coexistence of G6PD deficiency is noteworthy and merits cautious discussion. G6PD deficiency is characterized by impaired cellular defense against oxidative stress and is classically associated with increased susceptibility to hematologic injury under specific triggers [4]. In the setting of acute myocardial infarction, invasive treatment, and intensive antithrombotic exposure, this background condition may indicate increased hematologic vulnerability.

At the same time, the present case does not provide direct evidence that G6PD deficiency contributed to the onset or severity of thrombocytopenia. No laboratory markers of oxidative stress, hemolysis, platelet‐bound antibodies, or immune activation were available to support a specific mechanistic link. Accordingly, it is more appropriate to regard G6PD deficiency as a clinically relevant background condition rather than a proven mechanistic cofactor. The dominant explanation remains tirofiban‐associated thrombocytopenia itself, and whether G6PD deficiency modifies susceptibility to this complication cannot be determined from a single case [3, 4, 8].

### 3.3. Tirofiban Use and Acute Management

Another important issue concerns the indication for tirofiban in this patient. In contemporary practice, intravenous glycoprotein IIb/IIIa inhibitors are generally reserved for selected bailout situations during PCI, such as large thrombus burden, no‐reflow, or thrombotic complications, rather than used routinely [2]. In the present case, tirofiban was administered because coronary angiography demonstrated complete occlusion of the mid‐to‐distal right coronary artery with a large thrombus burden during primary PCI. Although formal thrombus grading was not prospectively documented, the procedural context supports its use as an intensified bailout antithrombotic strategy rather than standard routine treatment. This indication should nevertheless be described transparently, especially given the patient′s known G6PD deficiency and the need for individualized risk‐benefit assessment.

Once profound thrombocytopenia was identified, immediate withdrawal of the suspected culprit drug was the key therapeutic step. IVIG was administered because the platelet nadir was extremely low in a patient who had recently undergone PCI and remained exposed to both bleeding and ischemic risk. However, the role of IVIG should be interpreted cautiously. In drug‐induced immune thrombocytopenia, prompt discontinuation of the offending agent is the cornerstone of management, whereas IVIG is generally considered an adjunctive option in severe thrombocytopenia or in patients at high risk of bleeding [9]. Therefore, this case supports IVIG as a reasonable rescue measure in selected severe presentations, but not as evidence that recovery was necessarily attributable to IVIG itself.

### 3.4. Reinitiation of Antithrombotic Therapy and Possible Alternatives

The reinitiation of antithrombotic therapy after profound thrombocytopenia represented a major therapeutic challenge. The patient had recently undergone primary PCI for STEMI and remained at risk of recurrent coronary thrombosis, yet premature restoration of intensive antithrombotic therapy could have increased bleeding risk while platelet counts were still recovering. In this setting, sequential reintroduction of clopidogrel followed by indobufen was adopted as an individualized transitional strategy after platelet recovery had begun. This approach appeared feasible in the present patient, but it should not be generalized as an established safe regimen. Rather, it should be understood as a pragmatic case–based strategy implemented under close monitoring. Temporary P2Y12 inhibitor monotherapy was also a possible alternative; however, given the very recent primary PCI for STEMI and concern for early thrombotic risk, a stepwise reintroduction strategy was favored once platelet recovery became evident.

The potential role of cangrelor also deserves consideration. As a rapidly acting and reversible intravenous P2Y12 inhibitor, cangrelor may represent an alternative when short‐acting parenteral platelet inhibition is needed in the PCI setting. Recent pharmacodynamic data from the POMPEII Registry showed effective platelet inhibition in patients with acute or chronic coronary syndromes undergoing PCI, including those with STEMI [10]. However, the present case cannot establish that cangrelor would have been superior or safer in this specific clinical context, and this possibility should therefore be presented as a therapeutic consideration rather than a conclusion.

### 3.5. Clinical Implications

This case offers several practical lessons. First, profound thrombocytopenia occurring within hours after tirofiban exposure should prompt immediate consideration of acute glycoprotein IIb/IIIa inhibitor–associated thrombocytopenia [3, 8]. Second, when heparin has also been administered, differential diagnosis should be structured rather than assumption‐driven; a very low 4Ts score, very early onset, and absence of thrombosis together strongly argue against HIT [6, 7]. Third, in patients who have recently undergone PCI, management should extend beyond withdrawal of the suspected agent to include a carefully individualized plan for antithrombotic reinitiation [9].

This case also supports close platelet monitoring during the early period after tirofiban exposure, particularly when the drug is used in bailout situations with high thrombus burden. Early recognition of this rare but potentially life‐threatening complication may facilitate prompt drug withdrawal, more efficient differential diagnosis, and safer planning of subsequent antithrombotic therapy [3, 8].

## 4. Conclusions

We report a case of profound acute thrombocytopenia occurring shortly after tirofiban exposure during primary PCI in a patient with known G6PD deficiency. Based on the very early onset, absence of thrombosis, very low 4Ts score, and exclusion of other common causes, the overall clinicopathologic pattern was most consistent with acute tirofiban‐associated thrombocytopenia rather than HIT. Prompt discontinuation of the suspected agents, careful exclusion of alternative etiologies, and individualized reinitiation of antithrombotic therapy were central to the favorable outcome. Although the coexistence of G6PD deficiency is clinically noteworthy, its mechanistic contribution remains uncertain and should be clarified in future studies rather than inferred from a single case.

## Author Contributions

Chuanwei Zhao: conceptualization, clinical supervision, data interpretation, manuscript review and editing, and final approval. Yansi Wang: data collection, case management, and drafting of the manuscript. Yane Yang: data collection, literature review, and drafting of the manuscript. Shizhen Gao: clinical data acquisition, case management, and manuscript drafting. Wei Meng: clinical data collection and manuscript revision. Wenjie Tan: data verification and manuscript revision. Chuanwei Zhao, Yansi Wang, Shizhen Gao, and Wei Meng contributed equally to this work.

## Funding

No funding was received for this manuscript.

## Disclosure

All authors read and approved the final manuscript.

## Ethics Statement

Written informed consent for publication was obtained from the patient. The study was conducted in accordance with institutional ethical standards and the principles of the Declaration of Helsinki.

## Consent

The patient provided written informed consent for publication of the case details and accompanying figure.

## Conflicts of Interest

The authors declare no conflicts of interest.

## Data Availability

The data that support the findings of this study are available on request from the corresponding author. The data are not publicly available due to privacy or ethical restrictions.
